# Efficient Bayesian inference under the structured coalescent

**DOI:** 10.1093/bioinformatics/btu201

**Published:** 2014-04-20

**Authors:** Timothy G. Vaughan, Denise Kühnert, Alex Popinga, David Welch, Alexei J. Drummond

**Affiliations:** ^1^Allan Wilson Centre for Molecular Ecology and Evolution, Massey University, Palmerston North 4442, New Zealand, ^2^Institute of Integrative Biology, Swiss Federal Institute of Technology (ETH), Zurich 8092, Switzerland and ^3^Department of Computer Science, University of Auckland, Auckland 1142, New Zealand

## Abstract

**Motivation:** Population structure significantly affects evolutionary dynamics. Such structure may be due to spatial segregation, but may also reflect any other gene-flow-limiting aspect of a model. In combination with the structured coalescent, this fact can be used to inform phylogenetic tree reconstruction, as well as to infer parameters such as migration rates and subpopulation sizes from annotated sequence data. However, conducting Bayesian inference under the structured coalescent is impeded by the difficulty of constructing Markov Chain Monte Carlo (MCMC) sampling algorithms (samplers) capable of efficiently exploring the state space.

**Results:** In this article, we present a new MCMC sampler capable of sampling from posterior distributions over structured trees: timed phylogenetic trees in which lineages are associated with the distinct subpopulation in which they lie. The sampler includes a set of MCMC proposal functions that offer significant mixing improvements over a previously published method. Furthermore, its implementation as a BEAST 2 package ensures maximum flexibility with respect to model and prior specification. We demonstrate the usefulness of this new sampler by using it to infer migration rates and effective population sizes of H3N2 influenza between New Zealand, New York and Hong Kong from publicly available hemagglutinin (HA) gene sequences under the structured coalescent.

**Availability and implementation:** The sampler has been implemented as a publicly available BEAST 2 package that is distributed under version 3 of the GNU General Public License at http://compevol.github.io/MultiTypeTree.

**Contact:** tgvaughan@gmail.com

**Supplementary information:** Supplementary data are available at *Bioinformatics* online.

## 1 INTRODUCTION

Model-based phylogenetic inference has become one of the principal methods of using genetic sequence data to test scientific hypotheses pertaining to evolving populations. Its wide-spread adoption has been driven not only by improvements in computational and sequencing hardware but also by advances in methods of statistical inference. An important initial step in this development was the derivation of the *n*-coalescent process (Kingman, 1982), which probabilistically ties the shape of a rooted phylogenetic tree of *n* randomly chosen individuals to the parameters of an underlying Wright–Fisher population genetics model. In conjunction with Felsenstein’s ‘pruning algorithm’ (Felsenstein, 1981), this allowed for the computational inference of the sample genealogy itself alongside population genetic parameters, such as the effective population size.

Since these beginnings, an array of sophisticated models and inference methods has been developed, allowing molecular mutation rates, population size histories and other population genetic parameters of interest to be inferred alongside increasingly sophisticated phylogenetic tree reconstructions, which, for example, do away with the assumption of a strict molecular clock (Drummond *et al.*, 2006; Sanderson, 2002; Thorne *et al.*, 1998). Rapidly evolving populations such as viruses and bacteria are of particular interest, as such populations may undergo significant demographic variation over timescales comparable with the age of the genealogy of sampled data. Such data form the basis of the emerging field of phylodynamics (Grenfell *et al.*, 2004; Kühnert *et al.*, 2011; Lemey *et al.*, 2009; Volz *et al.*, 2013), which exploits these overlapping timescales by using the genetic data to select and infer parameters of sophisticated epidemiological models.

Many of the models that are of interest to population geneticists and epidemiologists are structured in some way. This structure may represent the spatial subdivision of a population into several distinct ‘demes’, or it may represent some other logical categorization of individuals such as the temporal subdivision used in compartmental epidemiological models. Such structure can strongly affect the shape of inferred genealogies (Pannell, 2003), and can provide a source of statistical bias when ignored.

A time-tested means of including this structure in phylogenetic analyses is through the use of the structured coalescent (Hudson, 1990; Notohara, 1990), an extension of Kingman’s coalescent in which the unstructured Wright–Fisher model is replaced by a structured equivalent encompassing a number of discrete subpopulations. Phylogenetic analyses based on this model are capable of both (i) reducing model misspecification bias in the inference of the genealogy and (ii) estimating parameters specific to structured models such as migration rates and effective subpopulation sizes. While similar, this model is distinct from the character-based treatment of lineage locations used by Lemey *et al.* (2009), in that the structured coalescent allows location to explicitly affect coalescent rate. (This is discussed in Section 1 of the Supplementary Material.)

At least two distinct Markov Chain Monte Carlo (MCMC) schemes for conducting inference under the structured coalescent exist in the literature. Both of these schemes use an MCMC algorithm to explicitly sample both the demographic and evolutionary model parameters and the structured genealogy: a phylogenetic tree annotated with individual migration events. The first of these schemes is the method of Beerli and Felsenstein (Beerli, 2006; Beerli and Felsenstein, 1999, 2001), which is implemented in the software package Migrate-n. At its core, this method involves a single proposal function, which updates the structured tree by ‘dissolving’ a randomly selected edge and drawing a new edge by simulating from the structured coalescent conditional on the remaining edges.

The other scheme is the work of Ewing *et al.* (2004), who have published a set of simple and fast proposal functions that act solely on the migration events on the structured genealogy. In combination with those for traversing the space of unstructured trees (Drummond *et al.*, 2002), Ewing et al. showed that an MCMC algorithm based on these moves is capable not only of jointly inferring the structured tree and migration model parameters, but also of exploiting the additional information contained in serially sampled data to infer absolute migration rates. While certainly functional and useful, this scheme suffers from performance issues, which arise in the form of slow ‘mixing’, meaning that MCMC calculations must be run for a long time to obtain useful information about the posterior probability distribution. An additional issue is that no implementation of the sampler has been made widely available.

In this article, we introduce a new set of MCMC proposal functions (or ‘operators’) that provide an efficient means of using serially sampled sequence data to infer the full structured tree and related model parameters (including mutation rates) under the structured coalescent. These operators, together with the data structure representing the structured tree itself and the probability density calculation algorithm for the structured coalescent, are implemented and distributed as a package extension to BEAST 2 (Bouckaert *et al.*, 2014; http://www.beast2.org). When applied to equivalent data, and assuming equivalent evolutionary models and parameter priors, this new package yields posterior distributions, which are exactly equivalent to those obtained using Migrate-n. However, the use of the BEAST 2 platform for our implementation gives our sampler access to a large array of molecular evolution models and parameter priors not yet available in Migrate-n. Additionally, we have implemented the operators described by Ewing et al., allowing direct comparison between the two sampling methods. This comparison shows that the new operators achieve significantly faster mixing when applied to simulated data, with an order of magnitude improvement in some cases. We go on to demonstrate the practicality of the new sampler by using it to infer mutation rates, effective population sizes and the structured tree from geographically annotated HA gene sequences derived from the Influenza A subtype H3N2 strain. We interpret these results in light of recent work by Bedford *et al.* (2010).

## 2 MATHEMATICAL BACKGROUND

### 2.1 Structured tree definition

Before discussing the inference procedure, we need to define precisely what we mean by a ‘structured tree’.

In this article, we define a structured tree T of *n* leaves as a fully resolved, rooted and timed phylogenetic tree in which every internal node represents a coalescent event and where every point on each edge of the tree is associated with exactly one type *d* drawn from a fixed set *D* of such types. Mathematically, we write T=(V,E,t,M). The first three elements are the usual phylogenetic tree components: a set *V* of 2n−1 nodes, a set *E* containing directed edges of the form 〈i,j〉 between nodes i,j∈V and a set of node ages $t=\{t_{i}|i\in V\}$ where *t_i_* is the age of node *i*. The direction of each edge 〈i,j〉 is such that $t_{i}<t_{j}$. The set of nodes is partitioned into two smaller sets *Y* and *I*, representing the *n* − 1 internal and *n* external nodes, respectively.

**Fig. 1.**
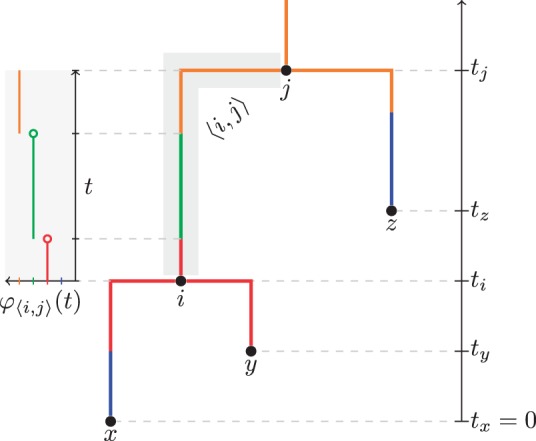
A structured tree T=(V,E,t,M) with V=I∪Y where I={x,y,z},Y={i,j}, E={〈x,i〉,〈y,i〉,〈i,j〉,〈z,j〉} and the coalescence times **t** and type mappings *M* are as shown. Here we have selected the type set D={blue,red,green,orange}, although this can be composed of the values of any discrete trait

The final element in T is the one that is unique to structured trees and is defined by $M=\{\varphi_{\langle i,j\rangle }|\langle i,j\rangle \in E\}$, where each function $\varphi_{\langle i,j\rangle }:[t_{i},t_{j}]\to D$ is piecewise constant and defined such that $\varphi_{\langle i,j\rangle }(t)$ is the type associated with the time *t* on edge 〈i,j〉∈E. Such a tree is illustrated in Figure 1.

We caution that the term ‘structured tree’ is not a standard name. For instance, elsewhere these objects are referred to as ‘migration-coalescent trees’ (Ewing *et al.*, 2004) or simply as ‘genealogies’ (Beerli and Felsenstein, 1999; Hudson, 1990). Our choice is based on the desire to at once distinguish these trees from regular phylogenetic trees and to extend their applicability beyond the special case of spatial structuring.

### 2.2 Bayesian inference framework

The goal of the inference scheme discussed here is to characterize the joint posterior probability density $P(E,t_{Y},M,\mu ,m,\theta |S,t_{I},L)$, where $t_{Y}=\{t_{i}|i\in Y\}$ and $t_{I}=\{t_{i}|i\in I\}$ are the times of the internal and external nodes, μ represents the set of substitution model parameters, *m* and θ are the immigration rate matrix and population size vectors defined later and $S=\{s_{i}|i\in I\}$ and $L=\{l_{i}|i\in I\}$ are the sequences and types associated with each of the leaf nodes. The values of $S,t_{I}$ and *L* represent the data.

The inference framework centres around the following expansion:
(1)
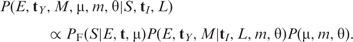



The first term $P_{F}(S|E,t,\mu )$ is the probability of the sequence alignment and can be efficiently evaluated using Felsenstein’s pruning algorithm (Felsenstein, 1981). The second term captures the joint probability of the genealogy and the type mapping, conditional on the number of samples, their types and the times at which they were recorded, and the demographic model parameters. This is given by the structured coalescent, as discussed below. The final term is the joint prior for all model parameters and can be factorized into P(μ)P(m)P(θ).

Our goal here is to use MCMC to draw samples from Equation (1), thus allowing us to uncover what the data have to say about the structured tree and the demographic and evolutionary model parameters.

### 2.3 The structured coalescent probability density

The structured coalescent, allowing for serially sampled sequences as detailed by Ewing *et al.* (2004), assumes the following demographic model. A discrete set of connected subpopulations *D* with sizes *N_d_* for d∈D are evolving under a Wright–Fisher model in which each generation of length *g* is divided into two stages. In the first stage, each haploid individual in the model migrates from its current location/type *d* to a new location *d′* with probability $q_{dd\prime }g$, or remains in the same subpopulation with probability $1-\sum_{d\prime \in D\setminus d}q_{dd\prime }g$. In the second stage, *N_d_* individuals are sampled with replacement from the occupants of each subpopulation following stage 1, with the sampled individuals forming the next generation.

To express the probability density of a structured tree under this model, we require the following additional definitions. First, we divide the period spanned by the tree into *B* adjacent intervals of lengths $\tau_{1},\tau_{2},\ldots ,\tau_{B}$ such that $\sum_{\alpha =1}^{B}\tau_{\alpha }=t_{r}$ where *t_r_* is the age of the root. Each interval is bracketed by a pair of consecutive ‘events’, each of which may be a ‘coalescent’, ‘migration’ or ‘sampling’ event. Coalescent events correspond to internal nodes i∈Y, migrations correspond to discontinuities in the type functions $\varphi_{\langle i,j\rangle }(t)$ and sampling events correspond to leaf nodes x∈I. The total number of migration events from the *d ′* type to the *d* type in *M* is given by $\nu_{dd\prime }^{m}$, while the total number of coalescent events (i.e. internal tree nodes) occurring in type *d* is $\nu_{d}^{c}$. Finally, the number of tree edges 〈i,j〉 for which $\varphi_{\langle i,j\rangle }(t)=d$ for *t* in interval α is given by $k_{\alpha ,d}$. All of this information is readily available from T as defined previously.

The probability density of the components of the structured tree before conditioning on the sequence data is then given by
(2)
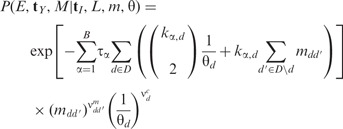

where $\theta_{d}=N_{d}g$ are population sizes scaled by the generation length and $m_{dd\prime }\equiv (\theta_{d}/\theta_{d\prime })q_{d\prime d}$ are the immigration rates into *d* from *d ′* (per individual in *d ′*). (This is essentially Equation (3) from Beerli and Felsenstein (2001) but allowing for heterochronous leaves.)

The structured coalescent, as well as other models including the reversible Markov model used by Lemey *et al.* (2009), imposes the following additional constraint on the structured tree beyond those laid out in section 2.1: that the type functions for all edges meeting at node *i* possess the same value at *t_i_*. Formally,

(3) $$\varphi_{\langle i,i_{p}\rangle }(t_{i})=\varphi_{\langle i_{cl},i\rangle }(t_{i})=\varphi_{\langle i_{cr},i\rangle }(t_{i})$$

where $i_{p},i_{cl}$ and *i_cr_* are the parent, left child and right child of *i*, respectively.

## 3 MCMC SAMPLING ALGORITHM

As outlined above, our goal here is to use MCMC to efficiently draw samples from the joint posterior Equation (1) over the state space spanned by $x=(E,t_{Y},M,\mu ,m,\theta )$. To perform efficiently, MCMC sampling algorithms need to be able to (i) rapidly calculate the value of a target distribution for a particular state *x* and (ii) propose new states *x ′* that are far enough from the current state for state space to be explored relatively quickly but not so far that the proposal acceptance rate becomes low.

Much of this problem is solved by existing methods. For reversible substitution processes, the probability of the sequence alignment given the tree can be efficiently evaluated using the pruning algorithm (Felsenstein, 1981). For inference under the structured coalescent, the density of the structured genealogy can be evaluated very simply by directly applying Equation (2), which scales according to the number of ‘events’ (coalescent, migration and sampling) making up the tree. The prior densities for the parameters (μ,*m*,θ) are usually chosen to be standard functions for which numerical evaluation is straightforward. In terms of state proposal, standard proposal distributions for sampling distributions over real numbers can be used to propose new parameter combinations.

The remaining component is a set of proposal operators that allow exploration of those regions of T space that have finite support under the structured coalescent. As noted above, this has been addressed in two distinct ways by Beerli and Felsenstein (1999) and Ewing *et al.* (2004). Here we introduce a novel set of operators that directly builds on an existing set of unstructured phylogenetic tree operators (Drummond *et al.*, 2002) that form the basis for the phylogenetics package BEAST (Bouckaert *et al.*, 2014; Drummond and Rambaut, 2007; Drummond *et al.*, 2012) and as such have been shown capable of efficiently traversing the space of $\left(E,t_{Y}\right)$.

### 3.1 Structured tree operator design strategy

Our general operator construction strategy involves the following:

1. the application of an existing unstructured tree operator, mapping $\left(E,t_{Y})\to (E\prime ,t_{Y}^{\prime }\right)$, followed by
2. the application of an edge type function proposal, which produces type functions $\varphi_{\langle i,j\rangle }\in M\prime$ for a subset of the edges 〈i,j〉∈E′ to ensure Equation (3) is satisfied at all nodes.

New type functions are proposed by using a backward-in-time continuous-time Markov chain (CTMC) to describe the type-change process along an edge, with the type transition rate matrix fixed to the jointly estimated structured coalescent immigration rate matrix *m*. We define the CTMC in terms of the probability of the edge taking a particular type given the type at the most recent node, $p(d,t)\equiv P(\varphi_{\langle i,j\rangle }(t)=d|\varphi_{\langle i,j\rangle }(t_{i})=d_{i})$, by way of the following master equation:

(4) $$\frac{d}{dt}p(d,t)=\sum_{d\prime \in D}m_{dd\prime }p(d\prime ,t)$$

where we define $m_{dd}=-\sum_{d\prime \in D\setminus d}m_{dd\prime }$.

Drawing type-space paths from Equation (4) is straightforward (Gillespie, 1976, 1977). However, to ensure Equation (3) is satisfied, we need to be able to draw paths from the CTMC conditioned on the type *d_i_* and *d_j_* at ‘both’ ends of an edge 〈i,j〉. To do this, we use the uniformization-based scheme of Fearnhead and Sherlock (2006). (A useful summary is provided by Rodrigue *et al.* (2008).)

This method involves defining a ‘uniformized’ version of the process that has a uniform intensity $\lambda =\max_{d}[-m_{dd}]$ over the length of an edge. This is accomplished by allowing for ‘do nothing’ transitions that are not associated with a type change. This means that the number of transition events and the time at which the events occur do not depend on exactly which transitions occur. We can therefore sample these aspects of the type function first.

The number of ‘virtual’ events *n_v_*, which includes the ‘do nothing’ transitions, is distributed according to

(5) $$P(n_{v}|d_{i},d_{j})=\frac{P(n_{v}){\left[R^{n_{v}}\right]}_{d_{i},d_{j}}}{{\left[e^{m\Delta }\right]}_{d_{i},d_{j}}}$$

where $\Delta =t_{j}-t_{i},P(n_{v})$ is a Poissonian with rate parameter λΔ,R is the stochastic matrix m/λ+I and $e^{m\Delta }$ is a matrix exponential. We sample from this distribution using the technique described in Section 2 of the Supplementary Material, then sample the event times by uniformly selecting *n_v_* times from the interval [*t_i_*,*t_j_*] and sorting the result.

The remaining problem of sampling the transition types themselves is equivalent to sampling paths of a discrete time Markov chain conditional on the end states, and is addressed using the standard forward–backward algorithm (Baum *et al.*, 1970).

One difficulty with this approach is its reliance on the matrix exponential $e^{m\Delta }$. Identifying general numerical approaches to matrix exponentiation is known to be problematic (Moler and Van Loan, 2003). We use the scaled Padé approximation method as implemented in the Java library jblas (http://mikiobraun.github.io/jblas), which generally performs well. However, in our experience it can become unreliable when very large and very small (yet non-zero) migration rates exist in the same matrix. For some data and prior combinations, it may therefore be necessary to temporarily switch to an alternative proposal mechanism when the MCMC chain strays into problematic regions.

### 3.2 Proposal acceptance probabilities

Non-unitary proposal acceptance probabilities are integral to the Metropolis–Hastings MCMC algorithm. For a given proposal operator op, a proposed state *x ′* is accepted with the probability

(6) $$\alpha_{\text{op}}(x\prime ,x)=\min\left(1,\frac{f(x\prime )}{f(x)}\rho_{\text{op}}(x\prime |x)\right)$$

where *f*(*x*) is the target density and $\rho_{\text{op}}(x\prime |x)$ is what we refer to as the Hastings–Green factor (HGF)—a generalization of the usual Hastings ratio to reversible jump MCMC operators (Green, 1995).

To calculate the HGF for each of our new operators, we need to be able to determine the probability density with which a particular type-change path is proposed. This density is given by

(7) $$P(\varphi_{\langle i,j\rangle }|d_{i},d_{j})=\frac{P(\varphi_{\langle i,j\rangle }|d_{i})}{P(d_{j}|d_{i})}$$

Here $P(\varphi_{\langle i,j\rangle }|d_{i})$ is the probability of the CTMC path conditional only on the most recent type, which can be derived directly from Equation (4) and is a simple product of exponential waiting time factors and transition rates. The denominator $P(d_{j}|d_{i})$ is the total transition probability over the length of the edge and may be computed using the same matrix exponential $e^{m\Delta }$ used in the previous section.

### 3.3 Structured tree operators

As described above, the tree-specific operators present in our sampler are straightforward extensions of the unstructured tree operators described by Drummond *et al.* (2002). They include the following:

- The ‘Wilson–Balding’ move (Wilson and Balding, 1998), which disconnects a subtree and reattaches it at a randomly chosen location on the rest of the tree. Our extension requires generating a type function for the new connecting edge.
- The ‘subtree exchange’ move, which chooses two subtrees and switches the points at which they connect to the rest of tree. Again, our extension requires generating a type function for each of the two new edges.
- A ‘node height shifting’ move, which repositions a randomly selected internal node by drawing from a uniform distribution between its oldest child and its parent. Our implementation randomly selects a new type for the selected node and then generates three new type functions—one for each of the connecting edges.
- A ‘tree height scaling’ move, which does not alter the topology but instead scales the age of each node by a randomly chosen factor. Our extension does not generate new type functions but merely scales the times of the type changes in the same way. This move is also used by Ewing *et al.* (2004).

In addition, we include a ‘node retype’ operator, which selects a new type for an internal node and generates new type functions for the connecting edges. (This is a special case of the node height shift move.)

**Fig. 2.**
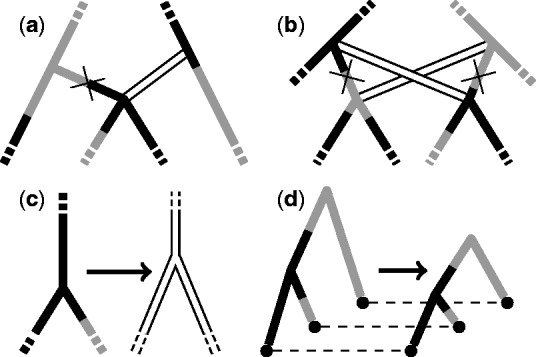
Schematics illustrating actions of the tree-specific operators used in our structured tree MCMC algorithm, including structured tree implementations of the (**a**) Wilson–Balding, (**b**) subtree exchange, (**c**) node height shift and (**d**) tree scaling operators. The solid edge shadings represent the deme to which each lineage belongs at each time. Double white lines represent edges for which new type functions will be proposed as part of the move, crosses represent edges to be removed and dashes represent edges that may continue beyond the schematic boundary

The actions of these operators are illustrated in Figure 2. For complete operator descriptions including HGFs, refer to Section 3 of the Supplementary Material.

## 4 IMPLEMENTATION

We have implemented the structured tree data structure, the structured coalescent tree density and the structured tree proposal operators as a BEAST 2 package. In this section we test the correctness and efficiency of the algorithm and its implementation.

### 4.1 Validation

In principle, a correctly implemented MCMC algorithm is capable of drawing samples from any target density or distribution defined over the state space traversed by its proposal operators. Thus, a good way to test correctness of an operator implementation is to use the implementation to produce a large number of samples from a target distribution that is either known exactly or can be sampled in some other independent way. Any disagreement between the MCMC-generated samples and the true distribution, or the externally generated samples from that distribution, is then indicative of an implementation error.

In our case, the structured coalescent density itself [Equation (2)] provides a sensible reference distribution, as its backwards-in-time Markovian structure allows structured trees to be easily sampled via stochastic simulation (Gillespie, 1976). The mean and variance of the root height are also known exactly for two taxon trees (see, for example, Hein *et al.*, 2005), allowing additional testing. Finally, this choice allows us to test the implementation of the structured coalescent density.

**Fig. 3.**
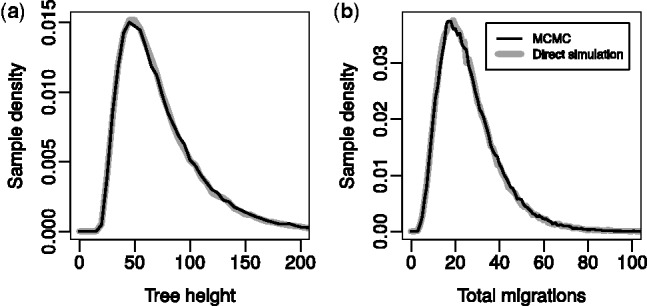
Agreement of (**a**) tree height and (**b**) migration count distributions sampled from the structured coalescent distribution using our implementation of the described MCMC algorithm (black lines) with those generated via direct simulation (grey lines). See text for more detail

Comparisons between the tree height and migration event counts obtained using our sampler (2 × 10^7^ steps minus 2 × 10^6^ burn-in) and those obtained through 10^5^ direct simulations generated using MASTER (Vaughan and Drummond, 2013) for trees with five leaves having times 0, 5, 10, 15, 20 and locations 0, 1, 2, 3, 0 in a 4 deme model with θ*_d_* = 7 and ${\text{m}}_{\text{dd}}^{\prime }$ = 0.05 for all *d*,*d ′* are shown in Figure 3. Additional comparisons for smaller sets of operators and against analytical results for the tree height expectations and variances are presented in Section 4 of the Supplementary Material. Together, these results are convincing evidence that the operators and structured coalescent density evaluation have been implemented correctly.

Note that using this package to perform inference from genetic data exploits additional machinery already present in the core BEAST 2 platform. In particular, modules for calculating the likelihood of a tree conditional on available sequence data are used. These modules have been implemented independently and have undergone their own extensive testing, so we do not explicitly test them here. (Indeed, this is one of the benefits of implementing a method using an existing inference platform.) However, many of those components are implicitly tested in the Migrate-n comparison reported below.

### 4.2 Inference from simulated data

We have applied the implemented sampler to the inference of evolutionary and demographic parameters from simulated sampling data to ensure that the inference scheme is capable of recovering the truth in situations where this is known *a priori*.

The data simulation procedure involved the following steps. (The relevant BEAST 2 XML files are provided as Supplementary Material.)

1. A structured coalescent model was chosen with a particular set of types, *D*, immigration rate matrix *m* and population size vector θ.
2. A 128 taxon structured coalescent tree was simulated under this model using MASTER, with times of the leaf nodes spread evenly among *t* = 0, 1, 2, 3 and the types of each set of 32 contemporaneous leaves chosen as evenly as possible from *D*.
3. A randomly selected 2 kb nucelotide sequence was evolved down this tree according to the HKY model (Hasegawa *et al.*, 1985) with transition/transversion rate ratio of 3 and base substitution rate μ_0_ = 0.005 subst./site/unit time using the BEAST 2 alignment simulator, resulting in a simulated alignment of 128 sequences.
4. The MCMC procedure outlined in the previous section was used to infer the parameters κ, μ_0_, *m* and θ, with log-normal prior distributions ln⁡N(0,4) on each element of these parameters. A total of 10^8^ MCMC steps were generated, with the first 10% being discarded to account for burn-in, resulting in an average effective sample size (ESS) of 1164 (5 and 95% sample quantiles 277 and 2425) for the slowest mixing parameter.

The diagrams in the first column of Table 1 illustrate three different structured coalescent models, with the nodes representing types *D*, the numeric labels on the nodes representing values of θ, the edges representing allowed transitions between these types and the numeric labels on the edges representing the values of *m*. The simulation and inference procedure was carried out 100 times for each of these models. The four right-most columns of the table present the fractions of inference runs that included the truth of the parameter at the head of the column within the 95% highest posterior density (HPD). In the case of θ and *m*, the average over each of the elements is displayed. Note that in the 4 deme mode, only the non-zero elements of *m* were used to calculate the coverage fraction although all elements were independently estimated.

**Table 1.** 95% HPD coverage fractions for demographic (population sizes θ, immigration rates *m*) and evolutionary parameters (clock rate μ_0_, transition/transversion ratio κ) inferred from simulated sequence data under structured population models with different numbers |*D*| of subpopulations

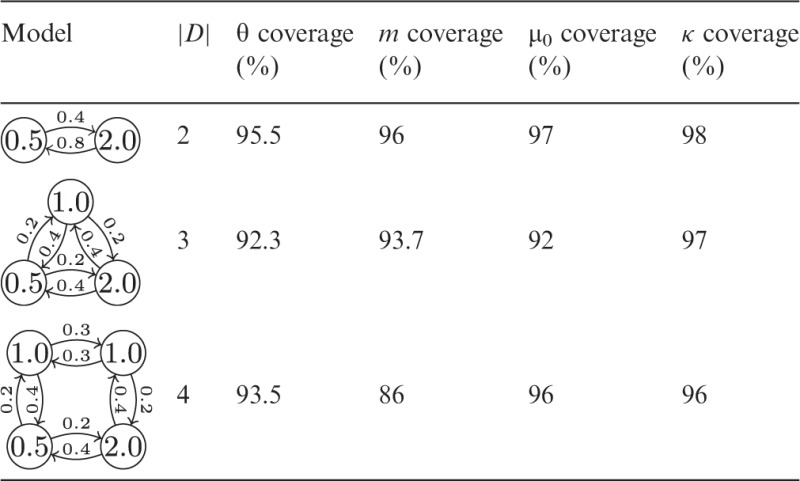

Section 5 of the Supplementary Material describes these results in more detail. An important message to convey here is that increasing the number of demes without increasing the available data can have strong negative effects on the amount of signal that can be recovered. For this reason, while the sampler itself is capable of sampling from structured tree distributions with higher numbers of demes (as shown in Section 5.2 of the Supplementary Material), doing so requires additional constraints on the model. In our experience, 3–4 demes seems to be an upper limit for inference from a 128 taxon dataset if all migration rates are to be reliably estimated (given non-informative priors).

### 4.3 Comparison with Ewing *et al.*, 2004

To compare the new structured tree operators with those of Ewing *et al.* (2004), we have implemented the operators described in that paper in our BEAST 2 package and used them to analyse the same simulated data sets described above. As each set of operators is capable of traversing the structured tree state space, there should be no difference in the inference results obtained by each set given sufficient MCMC steps. However, as discussed in Section 3, the specific proposal distributions used can have a significant impact on the rate at which the Markov chain produces effectively independent samples from the target distribution. This rate can be quantified using the inverse of the integrated auto-correlation time (IACT): the number of effectively independent samples (i.e. the ESS) generated per iteration of the MCMC algorithm. While IACT is usually expressed in terms of MCMC iterations, we use the total computation times used by each inference run to express it in terms of real time. This ‘effective sample rate’ (ESR) allows us to directly compare the computational efficiency of our proposal operators with those of Ewing *et al.* (2004), despite the fact that their operators are individually less computationally demanding. Such rates will depend on the specific hardware used to perform the computations, of course, but useful comparisons can still be made provided the same hardware is used across all analyses.

**Fig. 4.**
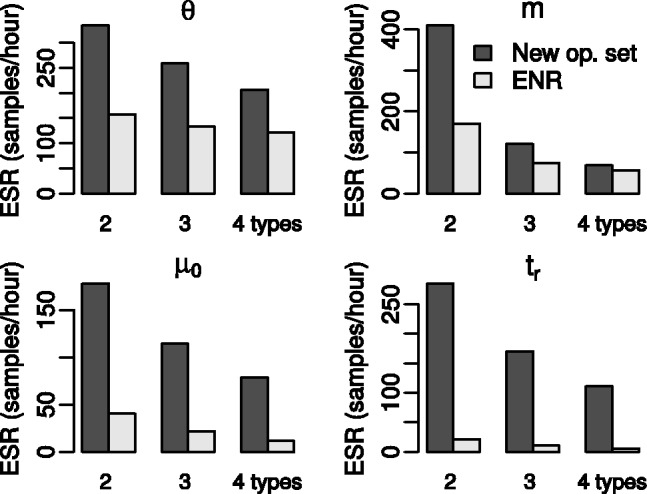
ESRs per hour of MCMC calculation recorded from the simulated data analyses using both the new proposal operators and our implementation of those developed by Ewing *et al.* (2004) (ENR), where θ is the vector of population sizes, *m* is the immigration rate matrix, μ_0_ is the clock rate and *t_r_* is the age of the root. Values for the vector/matrix parameters θ and *m* were averaged across all elements

Figure 4 shows how the ESRs for demographic (θ and *m*), evolutionary (μ_0_) and genealogy-related (time of most recent common ancestor, *t_r_*) parameters depend on the number of types, |D|, and the operator set used. Two features are immediately obvious. Firstly, there is a clear decline in ESR as the number of types increases, which is to be expected due to the corresponding increase in the size of the state space. Secondly, the ESR estimates obtained from the new proposal operators are greater than those obtained using our implementation of the operators proposed by Ewing *et al.* (2004). This improvement is particularly striking in the case of the *t_r_* parameter, for which our method produced at least an order of magnitude as many effective samples per unit time as our implementation of the previously published method.

We therefore find that the new operators presented here generally outperform our implementation of the Ewing *et al.* (2004) operators, in spite of the additional computational complexity of our proposals.

### 4.4 Comparison with Migrate-n

Migrate-n (Beerli and Felsenstein, 1999, 2001; Beerli, 2006) has long been a popular tool for performing both Bayesian and maximum-likelihood analysis under the structured coalescent. There are numerous differences between the MCMC sampler presented here and Migrate-n: we focus on providing the capability to perform joint estimation of heterochronous structured trees, demographic model parameters and substitution model parameters, while Migrate-n excels at demographic model parameter inference for trees with fixed clock rates or times expressed relative to an unknown substitution rate.

Despite this, it is possible to analyse some datasets under exactly equivalent assumptions in both packages. In Section 6 of the Supplementary Material, we directly compare sampled posteriors obtained for model parameters from heterochronous sequence datasets simulated under 2 and 3 deme models, assuming a known clock rate μ_0_, and find perfect agreement. Given the complete independence of these implementations, this is extremely strong evidence that both samplers are implemented correctly.

### 4.5 Application to global influenza epidemics

To assess the usefulness of our sampling strategy and its implementation in the context of real genetic data, we address the problem of inferring global dynamics of influenza epidemics from genetic data. To do this, we assembled 980 H3N2 1.6kb HA sequences from NCBI GenBank, which were isolated from humans in Hong Kong (*n* = 220), New York (*n* = 320) and New Zealand (*n* = 440) between the years 2000 and 2006. (Accession numbers, sampling times and locations are tabulated in the Supplementary Material.) These locations were chosen to be representative of northern, southern and equatorial regions having human population sizes of comparable order of magnitude, while the sampling time boundaries were chosen to ensure a roughly even temporal and spatial distribution of samples. Additionally, these locations and times allow comparison with the study of global H3N2 dynamics conducted by Bedford *et al.* (2010).

The data were analysed under a 3 deme structured coalescent model with a GTR+Γ nucleotide substitution model and a strict molecular clock. As for the simulated data, the heterochronous sampling times and rapid evolution of influenza allowed for joint estimation of the clock rate, μ_0_. Broad log-normal priors log⁡N(0,4) were used for all population size and rate parameters. Due to the large size of the data set, the MCMC algorithm was run for 3.7 × 10^8^ iterations to ensure adequate mixing, giving a ‘minimum’ ESS across the sampled parameters of ∼150. Two additional chains of 3.3 × 10^8^ iterations each were run to assess convergence. (See Section 7 of the Supplementary Material for details, the full set of results and ESS estimates.)

**Fig. 5.**
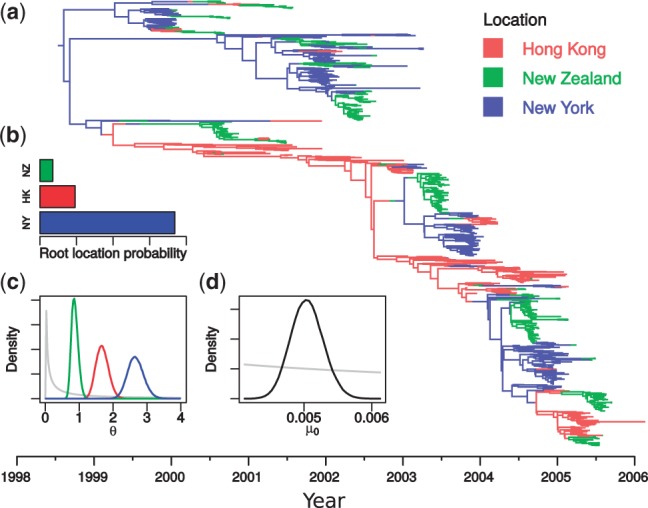
Summary of results from spatial H3N2 influenza analysis, including (**a**) the 980 taxon maximum sampled posterior structured tree, the sampled posterior probability distributions for (**b**) the root location, (**c**) the subpopulation sizes and (**d**) the base substitution rate (substitutions/site/year). The grey lines in (c) and (d) show the visible portions of the log⁡N(0,4) prior used for all of these parameters, scaled vertically for clarity

Figure 5 summarizes some of the results of this analysis, including the maximum sampled posterior tree, and posterior distributions for the root location, the subpopulation sizes θ and the molecular clock rate μ_0_. Assuming a constant generation time, the differences between elements of θ reflect differences in effective population sizes of the virus populations. It is therefore interesting that the order of these sizes corresponds to the ordering of the human population sizes: New Zealand (smallest), Hong Kong, New York (largest). Furthermore, the estimated base mutation rate $\mu_{0}≃5\times 10^{-3}$ substitutions/site/year [95% HPD interval $\left(4.5\times 10^{-3},5.5\times 10^{-3}\right)$] is in line with previous estimates for the HA gene (Rambaut *et al.*, 2008). The placement of the root of the maximum sampled posterior tree in New York is in agreement with the posterior probability distribution of the root location (Fig. 5b). This may seem contrary to conventional wisdom that Asia provides the source for seasonal influenza epidemics (Russell *et al.*, 2008). However, the location of the root is very much a function of the particular dataset used. Furthermore, our result is in line with the results of Bedford *et al.* (2010) who applied a hierarchical approach to a much larger dataset and found that the ‘trunk’ location of their sampled H3N2 transmission tree was likely also within the USA at the time of the root of our tree (mid 1998).

## 5 DISCUSSION

Taken together, the results above are strong evidence that our new algorithm and its implementation are correct, computationally efficient and capable of analysing large datasets. However, the algorithm presented here does not address problems that are fundamental to the structured coalescent. As discussed in detail by Ewing *et al.* (2004), the structured coalescent tree density [Equation (2)] can yield improper posterior densities for elements of the migration rate and population size parameters when uninformative priors are used. In our experience, this can still lead to slow mixing with proper but broad priors. In such cases, we suggest following the advice of Ewing *et al.* (2004) by imposing sensible upper and lower bounds on the demographic parameters whenever known.

There are many possible extensions to this work. Firstly, as we discuss in Section 1 of the Supplementary Material, other models involving structured trees exist beyond the structured coalescent. Some of these are likely to be particularly important in the field of viral phylodynamics, where the overlapping epidemiological and evolutionary time scales mean that birth–death sampling models (Stadler, 2008) or generalized coalescent processes (Volz, 2012) are needed to explain the sequence data. Developing, implementing and testing the performance of our proposal operators on spatial extensions to these models will therefore be an important area of future research.

Secondly, the problem of summarizing large numbers of structured trees sampled from posterior distributions requires special attention. While existing techniques for summarizing phylogenetic tree distributions [Felsenstein (2003) provides a good review] allow for the un-typed component of the trees in the sampled set to be summarized, this discards useful information. In our influenza analysis we chose to use the sampled structured tree with the HPD. While retaining type information, this approach is also suboptimal because it gives no indication of the uncertainty associated with the type-change paths depicted.

The BEAST 2 package implementing the algorithms discussed in this article and used in the analyses may be found at http://compevol.github.io/MultiTypeTree, together with an example analysis and a tutorial. The software source code is available under the GNU General Public License and is highly extensible, making third-party implementation of further structured tree models practical.

## Supplementary Material

Supplementary Data
