# Supplementary material for: Efficient Bayesian inference under the structured coalescent
Source: Bioinformatics. 2014 Apr 20;30(16):2272–9. doi: 10.1093/bioinformatics/btu201 (PMC4207426; doi:10.1093/bioinformatics/btu201)
Supplement: Supplementary Data [file supp_30_16_2272__index.html]

Efficient Bayesian inference under the structured coalescent — Efficient Bayesian inference under the structured coalescent — Efficient Bayesian inference under the structured coalescent — Supplementary Data 

# Efficient Bayesian inference under the structured coalescent

## Supplementary Data

files

**Files in this Data Supplement:**

- Supplementary Data - zip file
