# Supplementary material for: Efficient Bayesian inference under the structured coalescent
Source: Bioinformatics. 2014 Apr 20;30(16):2272–9. doi: 10.1093/bioinformatics/btu201 (PMC4207426; doi:10.1093/bioinformatics/btu201)
Supplement: Supplementary Data [file supp_btu201_suppl_data.zip › Supplementary.pdf]

Supplementary material for "Efficient Bayesian inference under the structured coalescent"

T.G. Vaughan, D. Kühnert, A. Popinga, D. Welch and A.J. Drummond

1 Relationship to other structured population models

In an ideal Bayesian context, inference of structured trees is accomplished by probing the joint probability density

$$P(E, \mathbf{t}_Y, M, \Theta | S, \mathbf{t}_I, L) \tag{1}$$

where E and M encompass the tree topology and migration paths as defined in the manuscript, $\mathbf{t}_Y = \{t_i | i \in Y\}$ are the times of the internal nodes, and Θ represents the full set of model parameters, conditional on the sequence alignment S, the times $\mathbf{t}_I = \{t_i | i \in I\}$ and types $L = \{l_i | i \in I\}$ associated with the sequences. With the density written in this unified form, the assumed model can include arbitrarily complex interplay between population dynamics and genotype, including the effects of natural selection. However, performing inference under such models is computationally demanding due to the amount of state augmentation required.

In statistical phylogenetics, we therefore often assume a simple stochastic model for sequence evolution and impose the condition of strict neutrality. This allows us to write

$$P(E, \mathbf{t}_Y, M, \Theta | S, \mathbf{t}_I, L) \propto P_{\mathrm{F}}(S | E, \mathbf{t}, \mu) P(E, \mathbf{t}_Y, M | \mathbf{t}_I, L, \bar{\Theta}) P(\Theta). \tag{2}$$

Here $P_F(S|E, \mathbf{t}, \mu)$ is the probability of the sequence alignment given the genealogy and the parameters μ of the sequence evolution model, and for many such models can be efficiently evaluated using Felsenstein's pruning algorithm [Felsenstein, 1981]. The second term captures the joint probability of the genealogy and the type mapping, conditional on the number of samples, their types and the times at which they were recorded, and the demographic model parameters $\bar{\Theta}$. The final term $P(\Theta)$ describes the joint prior for all model parameters $\Theta = \mu \cup \bar{\Theta}$.

When inferring unstructured trees (i.e. ordinary phylogenetic time trees), the second term in the expansion above is often chosen [Felsenstein, 2003] to

be the density for Kingman's n-coalescent process. This corresponds to a single panmictic population of fixed or deterministically varying size evolving under a neutral Wright-Fisher or similar (eg. Moran) model. A somewhat equivalent procedure for structured tree inference is to assume that $P(E, \mathbf{t}_Y, M|\mathbf{t}_I, L, \bar{\Theta})$ is given by the structured coalescent genealogical process [Hudson, 1990, Notohara, 1990]. This is obtained by applying Kingman's logic to a Wright-Fisher model incorporating multiple sub-populations of distinct but fixed sizes and connected by random and (in general) asymmetric migration.

Another popular model comparable to the structured coalescent has been developed by Lemey et al. [2009]. This involves an additional factorization of $P(E, \mathbf{t}_Y, M|\mathbf{t}_I, L, \bar{\Theta})$ under the assumption that the type of an individual has no bearing on its contribution to the demographic process. (In other words, the location of an individual has no bearing on the number of offspring it is likely to produce—in contrast to the Wright-Fisher model assumed by the structured coalescent process, where the maximum number of progeny of an individual is given by the population size in its deme.) This additional restriction allows the location to be treated as a special character in the genetic sequence evolving under its own substitution model. As such, it permits efficient marginalization over M using Felsenstein's pruning algorithm.

As noted in section 1 of the manuscript, population structure isn't necessarily the result of spatial segregation. The binary state speciation and extinction (BiSSE) model of Maddison et al. [2007], for instance, provides a probability density over structured trees where the (two) types represent the presence or absence of some trait that affects the fitness of an individual. Like the structured coalescent (and unlike the phylogeographic model of Lemey et al. [2009]), the BiSSE structured tree density cannot be factorized into the product of an unstructured tree density and the probability of the structuring. However, it is important to note that trait evolution models (and structured tree models in general) are not necessarily subject to the constraint that the types on the edges at an internal node must be equal (equation 3 in the manuscript).

2 Virtual event count sampling

We sample the number of virtual events under the uniformized CTMC process along an edge $\langle i, j \rangle$ of length $\Delta = t_j - t_i$ conditional on the types at the nodes, d_i and d_j, using a relatively straight-forward modification to the inversion sampling algorithm described by Rodrigue et al. [2008].

1. Draw u from Unif$(0, 1)$.
2. Set $n \leftarrow 0$ and $a \leftarrow 0$.
3. Evaluate R^n.
4. If $n >= 1$ and $R^n = R^{n-1}$ to machine precision, then set $C \leftarrow n$ and rejection sample n from Pois$(\lambda\Delta)$ conditional on $n >= C$. Return this value of n.

5. Set $a \leftarrow a + e^{-\lambda\Delta}(\lambda\Delta)^n[R^n]_{d,d'}/n!$.

6. If $a > u$ then return n, otherwise set $n \leftarrow n + 1$ and go to 2.

In contrast to the inversion sampling algorithm, this algorithm can efficiently deal with transition probability matrices that give rise to very large numbers of transitions. On the other hand, in contrast to a plain rejection sampling algorithm where one samples from Pois($\lambda\Delta$) and rejects based on $[R^n]_{d_i,d_j}$, this algorithm does not necessarily become inefficient when the n which maximizes $[R^n]_{d_i,d_j}$ is significantly different to $\lambda\Delta$.

We tested this algorithm and its implementation by comparison with an implementation of the unmodified cumulative algorithm of Rodrigue et al. [2008].

2.1 Explanation of algorithm

The algorithm above is centred around the idea of dividing the non-negative integers into $n_v < C$ and $n_v \geq C$. While the value of C is not known when the algorithm begins, it is a deterministic function of the stochastic matrix R and thus has a single well defined value. This value is chosen so that R^{n_v} is constant (to machine precision) for $n_v \geq C$, meaning that $P(n_v|a, b, n_v \geq C) \propto P(n_v)H(n_v - C)$ where $P(n_v)$ is a Poissonian with rate parameter $\lambda\Delta$ and $H(q)$ is the unit step function.

We use this cut-off to express the virtual event count probability in the following way:

$$
\begin{aligned}
P(n_v|a, b) &= P(n|a, b, n_v < C)P(n_v < C|a, b) + P(n|a, b, n_v \geq C)P(n_v \geq C|a, b) \\
&= P(n_v|a, b, n_v < C)P(n_v < C|a, b) + (P(n_v)H(n_v - C)/Z)P(n_v \geq C|a, b)
\end{aligned}
$$

where the normalization Z is constant with respect to n_v.

One way of using C to sample from n would be to first sample from the Boolean distribution $P(n < C|a, b)$ then, conditional on this result, use either

1. a cumulative distribution function approach to sample from the distribution $P(n_v|a, b, n_v < C)$, or

2. a standard Poissonian sampling algorithm to draw values from $P(n_v)$ until an n_v is drawn satisfying $n_v \geq C$.

This is essentially what the algorithm above does. However, rather than explicitly sampling from $P(n < C|a, b)$ and using the cumulative algorithm to sampling from $P(n_v|a, b, n_v < C)$, we combine these steps into one by instead using the cumulative algorithm to sample from $P(n_v|a, b)$ and switching to the Poissonian sampling algorithm when the cumulative algorithm fails to choose n_v satisfying $n_v < C$. We thus avoid having to explicitly calculate $P(n_v < C|a, b)$.

3 Complete MCMC operator descriptions

As described in the manuscript, the novel operators present in our sampler are straightforward extensions of the untyped tree operators described in detail by Drummond et al. [2002]. Here we detail each move and specify the way in which it is augmented using our edge type proposal scheme. In this section,

- $\mathcal{T}$ and $\mathcal{T}'$ are the original and proposed structured trees, respectively,
- $\text{Par}(i;\mathcal{T})$ is the parent of coalescent node i in $\mathcal{T}$,
- $\text{CL}(i;\mathcal{T})$ is the left child of node i in $\mathcal{T}$,
- $\text{CR}(i;\mathcal{T})$ is the right child of node i in $\mathcal{T}$, and
- $\text{Sib}(i;\mathcal{T})$ is the sibling of node i in $\mathcal{T}$.

Additionally, the notation $a \vee b$ is used to refer to the maximum of a and b.

3.1 Wilson-Balding

This operator affects the topology of the genealogy and involves three steps. Firstly, a pair of non-root nodes i and j are selected uniformly at random from V, with the move being rejected under certain conditions (such as i and j being too closely related or i being older than a parent of j).

Secondly, in the case that $i_p = \text{Par}(i;\mathcal{T})$ is not root, the edges between $i_s = \text{Sib}(i;\mathcal{T})$, i_p and $i_{pp} = \text{Par}(i_p;\mathcal{T})$ are replaced in E' with a single edge $\langle i_s, i_{pp}\rangle$ and the type functions $\varphi_{\langle i_s,i_p\rangle}$ and $\varphi_{\langle i_p,i_{pp}\rangle}$ in M are combined into a function $\varphi_{\langle i_s,i_{pp}\rangle} \in M'$. On the other hand, if i_p *is* the root of $\mathcal{T}$, the edge $\langle i_s, i_p\rangle$ in E and the corresponding type function $\varphi_{\langle i_s,i_p\rangle}$ in M are removed, and i_s becomes the root of $\mathcal{T}'$.

Thirdly, if j is not the root of $\mathcal{T}$, the time t'_{i_p} is chosen uniformly at random from the interval $[t_i \vee t_j, t_{j_p}]$ and the edge $\langle j, j_p\rangle$ in E where $j_p = \text{Par}(j;\mathcal{T})$ is replaced by edges $\langle j, i_p\rangle$ and $\langle i_p, j_p\rangle$. The corresponding type function $\varphi_{\langle j,j_p\rangle}$ is divided in two at t'_{i_p}, becoming $\varphi'_{\langle j,i_p\rangle}$ and $\varphi'_{\langle i_p,j_p\rangle}$. If j *is* the root of $\mathcal{T}$, the time t'_{i_p} is chosen from an exponential distribution of some mean α above t'_j, a new root type d_r is chosen uniformly from D and a new type function $\varphi'_{\langle j,i_p\rangle} \in M'$ is drawn under the condition that $\varphi'_{\langle j,i_p\rangle}(t'_{i_p}) = d_r$. In both cases, a new type function $\varphi'_{\langle i,i_p\rangle} \in M'$ is also generated, under the condition that $\varphi'_{\langle i,i_p\rangle}(t_i) = \varphi_{\langle i,i_p\rangle}(t_i) = d_i$ and $\varphi'_{\langle i,i_p\rangle}(t'_{i_p}) = \varphi'_{\langle j,i_p\rangle}(t'_{i_p}) = d'_{i_p}$.

The HGF $\rho_{\text{wb}}(x'|x)$ for this move depends on the precise variant of the move employed, as governed by the choice of i and j. In the case that neither i_p nor j is root in $\mathcal{T}$ we have

$$\rho_{\text{wb}}(x'|x) = \frac{t_{j_p} - (t_i \vee t_j)}{t_{i_{pp}} - (i \vee i_s)} \frac{P(\varphi_{\langle i,i_p\rangle}|d_i, d_{i_p})}{P(\varphi'_{\langle i,i_p\rangle}|d_i, d'_{i_p})} \quad (3)$$

while if i_p is root in $\mathcal{T}$ we have

$$\begin{aligned}\rho_{\mathrm{wb}}(x'|x) =&\alpha^{-1}\exp[-(t_{i_p}-t_{i_s})/\alpha](t_{j_p}-(t_i\vee t_j))\\ &\times\frac{P(\varphi_{\langle i,i_p\rangle}|d_i,d_{i_p})P(\varphi_{\langle i_s,i_p\rangle}|d_{i_s},d_{i_p})}{|D|P(\varphi'_{\langle i,i_p\rangle}|d_i,d'_{i_p})}.\end{aligned}\tag{4}$$

Finally, if j is root in $\mathcal{T}$ we have

$$\begin{aligned}\rho_{\mathrm{wb}}(x'|x) =&\alpha(\exp[-(t'_{i_p}-t_j)/\alpha](t_{i_{pp}}-(t_i\vee t_s)))^{-1}\\ &\times\frac{|D|P(\varphi_{\langle i,i_p\rangle}|d_i,d_{i_p})}{P(\varphi'_{\langle i,i_p\rangle}|d_i,d'_{i_p})P(\varphi'_{\langle j,i_p\rangle}|d_j,d'_{i_p})}.\end{aligned}\tag{5}$$

3.2 Subtree Exchange

This operator also alters the tree topology, but in a simpler way. It has two variants, "narrow" and "wide". In the narrow variant, a node i is chosen uniformly from V excluding the root and children of the root. We then define $i_p = \mathrm{Par}(i;\mathcal{T})$, $j = \mathrm{Sib}(i_p;\mathcal{T})$ and $j_p = \mathrm{Par}(j;\mathcal{T})$. The operator proceeds by replacing edges $\langle i, i_p\rangle$ and $\langle j, j_p\rangle$ in E with edges $\langle i, j_p\rangle$ and $\langle j, i_p\rangle$ in E'. Similarly, type functions $\varphi_{\langle i,i_p\rangle}$ and $\varphi_{\langle j,j_p\rangle}$ in M are replaced with new type functions $\varphi'_{\langle i,j_p\rangle}$ and $\varphi'_{\langle j,i_p\rangle}$ in M'. These new type functions are drawn from $P(\varphi_{\langle i,j_p\rangle}|d_i, d_{j_p})$ and $P(\varphi_{\langle j,i_p\rangle}|d_j, d_{i_p})$ using the algorithm described above, where d_i, d_j, d_{i_p} and d_{j_p} are the original types these nodes in $\mathcal{T}$.

The wide variant differs only in the way in which nodes i and j are chosen. In this case, i is chosen uniformly from V excluding only the root (not children of the root). Most importantly, however, j, is also drawn uniformly from the remaining nodes in V excluding the root and $\mathrm{Sib}(i;\mathcal{T})$.

The HGF for both variants of this move is

$$\rho_{\mathrm{sx}}(x'|x) = \frac{P(\varphi_{\langle i,i_p\rangle}|d_i,d_{i_p})P(\varphi_{\langle j,j_p\rangle}|d_j,d_{j_p})}{P(\varphi'_{\langle i,j_p\rangle}|d_i,d_{j_p})P(\varphi'_{\langle j,i_p\rangle}|d_j,d_{i_p})}.\tag{6}$$

3.3 (Joint) Tree Scaling

This operator does not affect the tree topology, leaving $E' = E$. It has two forms: in the first, a scaling factor f is chosen uniformly from the interval $[\beta^{-1}, \beta]$, and used to set $\mathbf{t}'_Y = f\mathbf{t}_Y$. Similarly, we set $\varphi'_{\langle i,j\rangle}(t) = \varphi_{\langle i,j\rangle}(t/f)$ for all $\langle i, j\rangle \in E$. As the elements of $\mathbf{t}_I$ are fixed, the scaling operation has the potential to result in node heights or type change times in $\mathcal{T}'$ which fall below the age of one or more descendant child nodes. In such cases, the proposal is rejected. The HGF for the scaling operator is

$$\rho_{\mathrm{ts}}(x'|x) = f^{C-2}\tag{7}$$

where we define $C = \sum_{d\in D}(\nu^c_d + \sum_{d'\in D}\nu^m_{d'd})$, borrowing notation from section 2.3 of the manuscript.

In the second form, parameters μ, m and θ are scaled jointly with the coalescent and type change event times. Specifically, the clock rate μ_0 and each of the migration rate matrix elements $m_{dd'}$ are scaled by f^{-1}, while the population sizes N are scaled by f. In this case, the HGF is

$$\rho_{\text{jts}}(x'|x) = \rho_{\text{ts}}(x'|x) f^{2|D|-|D|^2-1} \tag{8}$$

where $|D|$ is the number of types or demes in the model.

Unlike the others, this operator is not in any way novel and has been described previously by Ewing et al. [2004].

3.4 Node retype and shift-retype

This operator also has two variants. In the first, a node $i \in Y$ and a type $d_i' \in D$ are each selected randomly. In the following, we use $i_{cl} = \text{CL}(i;\mathcal{T})$, $i_{cr} = \text{CR}(i;\mathcal{T})$ and $i_p = \text{Par}(i;\mathcal{T})$. The type functions corresponding to all edges attached to i (edges $\varphi_{\langle i_{cl},i\rangle}$,$\varphi_{\langle i_{cr},i\rangle}$ and $\varphi_{\langle i,i_p\rangle}$ in the case that i is not the root) are then regenerated conditional on the original types $d_{i_{cl}} = \varphi_{\langle i_{cl},i\rangle}(t_{i_{cl}})$, $d_{i_{cr}}$ and d_{i_p} and the new type d_i at i. The HGF for the node retype operator is

$$\rho_{\text{nr}}(x'|x) = \frac{P(\varphi_{\langle i_{cl},i\rangle}|d_{i_{cl}}, d_i)P(\varphi_{\langle i_{cr},i\rangle}|d_{i_{cr}}, d_i)}{P(\varphi'_{\langle i_{cl},i\rangle}|d_{i_{cl}}, d_i')P(\varphi'_{\langle i_{cr},i\rangle}|d_{i_{cr}}, d_i')} \tag{9}$$

with an additional factor of $P(\varphi_{\langle i,i_p\rangle}|d_i, d_{i_p})/P(\varphi'_{\langle i,i_p\rangle}|d_i', d_{i_p})$ in the case that i is not root.

The second variant combines the retyping operations with alteration of the height of node i. For non-root i, the new node height t_i' is chosen uniformly from the interval $[(t_{i_{cl}} \vee t_{i_{cr}}), t_{i_p}]$ and $\rho_{\text{nsr}}(x'|x) = \rho_{\text{nr}}(x'|x)$. For root i we set $t_i' = t_c + f(t_i - t_c)$ with $t_c \equiv t_{i_{cl}} \vee t_{i_{cr}}$ and f drawn uniformly from $[\beta^{-1}, \beta]$. In that case, $\rho_{\text{nsr}}(x'|x) = \rho_{\text{nr}}(x'|x) f^{-1}$.

Note that incorporating a shift-retype move that *only* influences the root can improve mixing in some cases.

4 Implementation validation

In order to assess the correctness of the implementation of each operator and the structured coalescent density evaluator, the general approach is to use the MCMC algorithm implementation to sample from the structured coalescent itself conditional on a variety of fixed rate matrices m and population size vectors θ. Each set of samples is then tested against analytical results or samples generated independently using the direct simulation algorithm implemented in MASTER [Vaughan and Drummond, 2013].

The BEAST 2 XML input files specifying both the MCMC and direct simulation calculations employed in this section are available as part of the supplementary material in the archive file `ValidationScripts.zip`.

4.1 Comparison with analytical results

Here we exploit the fact that the mean and variance of the time to coalescence for two contemporaneously-sampled lineages in a model with $|D|$ demes, a uniform immigration rate matrix $m_{dd'} = \bar{m}$ and a uniform population distribution $\theta_d = \bar{\theta}$ are known exactly. (See, for example, Hein et al. [2005].) In such a model, the expected coalescence time is

$$E(T_{\text{same}}) = \bar{\theta}|D| \tag{10}$$

when the lineages are sampled from the same deme, and

$$E(T_{\text{diff}}) = \bar{\theta}|D| + \frac{1}{2\bar{m}} \tag{11}$$

when they are sampled from different demes. The corresponding variances are

$$\text{Var}(T_{\text{same}}) = (\bar{\theta}|D|)^2 + (|D|-1)\frac{\bar{\theta}}{\bar{m}} \tag{12}$$

and

$$\text{Var}(T_{\text{diff}}) = (\bar{\theta}|D|)^2 + (|D|-1)\frac{\bar{\theta}}{\bar{m}} + \frac{1}{(2\bar{m})^2}. \tag{13}$$

We used all of the proposal operators compatible with two-taxon trees (tree scaler, node retype and node shift-retype) in two MCMC runs with 10^7 steps, 10^6 of which were removed to allow for burn-in. Both runs sampled from an isochronous two-taxon structured coalescent distribution with $|D| = 4$ demes, $\bar{\theta} = 7$ and $\bar{m} = 0.05$. In the first run, both leaves were given the same type, while in the second run they were given different types.

The table below compares the structured coalescent tree height mean and variance estimates obtained using the MCMC algorithm with the exact analytical results. The uncertainty estimates are twice the standard deviation of each sample mean and variance, with the ESS of the MCMC chain used as the sample number.

Quantity	Analytical	MCMC
$E(T_{\text{same}})$	28	28.04 ± 0.35
$E(T_{\text{diff}})$	38	38.154 ± 0.25
$\text{Var}(T_{\text{same}})$	1204	1174 ± 34
$\text{Var}(T_{\text{diff}})$	1304	1289 ± 25

We thus find that our implementation of the described MCMC algorithm is consistent with the known exact results for these quantities.

4.2 Comparison with independent simulation results

Going beyond this simple comparison requires a numerical method of sampling from the structured coalescent that independent of the described MCMC algorithm. Since the structured coalescent can be regarded as a continuous-time

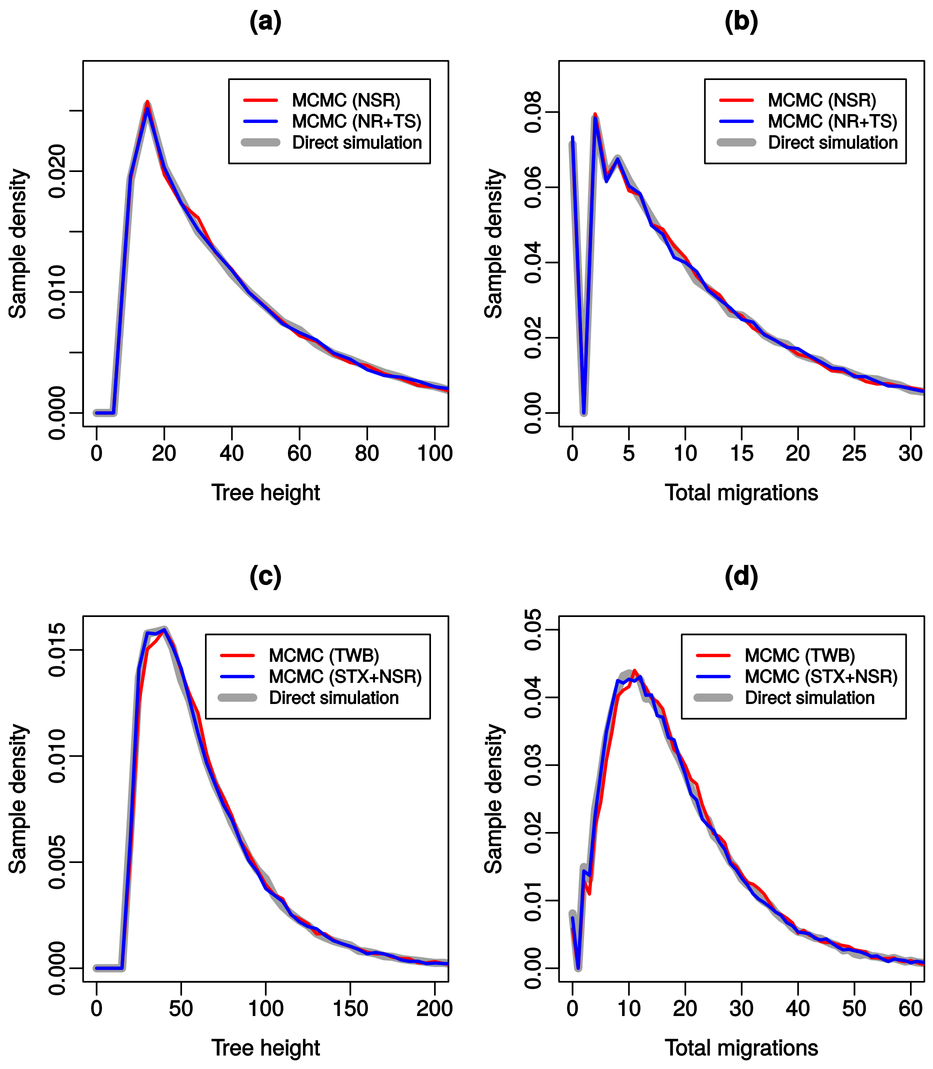

Figure 1: Comparisons between sampled distributions generated using the MCMC algorithm discussed in the manuscript and those generated using direct simulation. Sub-figures (a) and (b) respectively show the comparisons for the two taxon tree height and migration event count distributions for both the node shift-retype (NSR) and the node retype with tree scaling (NR+TS) MCMC calculations. Sub-figures (c) and (d) show the comparisons for the three taxon tree height and migration event count distributions for the typed Wilson-Balding (TWB) and the subtree exchange and node shift-retype (STX+NSR) calculations.

Markov process, the direct stochastic simulation algorithm of Gillespie [1976] is such a method. We use the implementation of this method found in MASTER [Vaughan and Drummond, 2013]. (Note that although that implementation is also technically a BEAST 2 package, there is no overlap between the stochastic simulation algorithm it implements and the structured coalescent MCMC algorithm implementation we are testing here.)

To test each operator in a situation as close to isolation as possible, we note that the following operator combinations are capable of exploring the space of heterochronous structured trees with the given numbers of leaf nodes:

- Trees with 2 leaves:
  - Node shift-retype in isolation
  - Node retype and structured tree scaler in combination
- Trees with 3 leaves:
  - Wilson-Balding in isolation
  - Subtree-exchange (narrow) and node shift-retype in combination

For each of these four operator combinations, 5×10^7 MCMC steps were generated with the first 5×10^6 omitted to allow for burn-in. In the two leaf cases, the age of one leaf was set to be 10 time units older than the other. In the three leaf cases, the ages of the leaves were set at 0, 10 and 20 time units, respectively. In all cases the leaf nodes were assumed to have the same type. In terms of the model, the number of demes was fixed at $|D| = 4$ with the migration rate matrix elements set uniformly to $m_{dd'} = 0.05$ and the scaled population size vector elements fixed to $\theta_d = 7$.

Figure 1 compares the sampled tree height densities and total migration count distributions generated using each of the operator combinations with distributions obtained from 10^5 direct MASTER simulations under the same model. While there is visible noise in the MCMC-derived distributions due to the slower mixing caused by the use of small operator sets, there is a clear agreement between the two sets of sample distributions in each case.

5 Inference from simulated data

The MASTER and BEAST 2 XML input files used to generate and perform inference under the simulated data in this section are provided in the supplementary file `SimulationInferenceScripts.zip`.

5.1 Full inference for 2–4 demes

Figure 2 displays the demographic and evolutionary parameter posterior densities obtained using our BEAST 2 package to analyze the sequence data simulated under the model shown in Table 1 of the manuscript. Note the significant difference between the priors (black lines) and the sampled densities, indicating that the data are highly informative in this case. A summary of the results for all analyses is shown in Table 1.

5.2 Conditioned inference for 5 and 10 demes

Performing inference under models assuming larger numbers of demes poses a problem for structured coalescent-based inference due to the fact that the total number of parameters in the model scales with the square of the number of demes, i.e. $|D|^2$. However, we demonstrate here that our sampler can cope with models having more than 4 demes when additional information counters this parameter explosion.

To do this, we generate two sets of 100 simulated alignments. In the first set, we assume a structured coalescent model with $|D| = 5$ demes having the scaled population size vector $\theta = (0.5, 1, 1, 1, 2)$ and a immigration rate matrix m with elements set uniformly to 0.05. MASTER was used to simulate 100 trees under this model, each having 50 leaves distributed evenly among times $t = 0, 1, 2, 3, 4$ and the 5 locations. A sequence was then simulated down each of these trees, again assuming an HKY substitution model with transition/transversion ratio $\kappa = 3$ and clock rate $\mu_0 = 0.005$, leaving 100 distinct simulated sequence alignments.

For the second data set, we assumed a model with $|D| = 10$ demes and a population size vector $\theta = (0.5, 0.5, 0.5, 1, 1, 1, 1, 2, 2, 2)$ and a uniform immigration matrix m with elements again set uniformly to 0.05. MASTER was again used to simulate 100 trees under the model, this time with 100 leaves but again distributed evenly among times $t = 0, 1, 2, 3, 4$ and each of the 10 locations. The sequence simulation then proceeded exactly as for the 5 deme case.

For each of these 200 simulated alignments, our structured coalescent sampler was used to generate posterior distributions for the structured tree, the substitution model parameters and the demographic model parameters. In the case of the demographic model, however, we exploited our knowledge that the immigration rate matrix elements were set to be equal by preventing the sampler from individually modifying single rate elements during the analyses. In both sets of analyses, log-normal priors $\log \mathcal{N}(0, 4)$ were used for the substitution model parameters, while $1/x$ priors were applied to the population size and

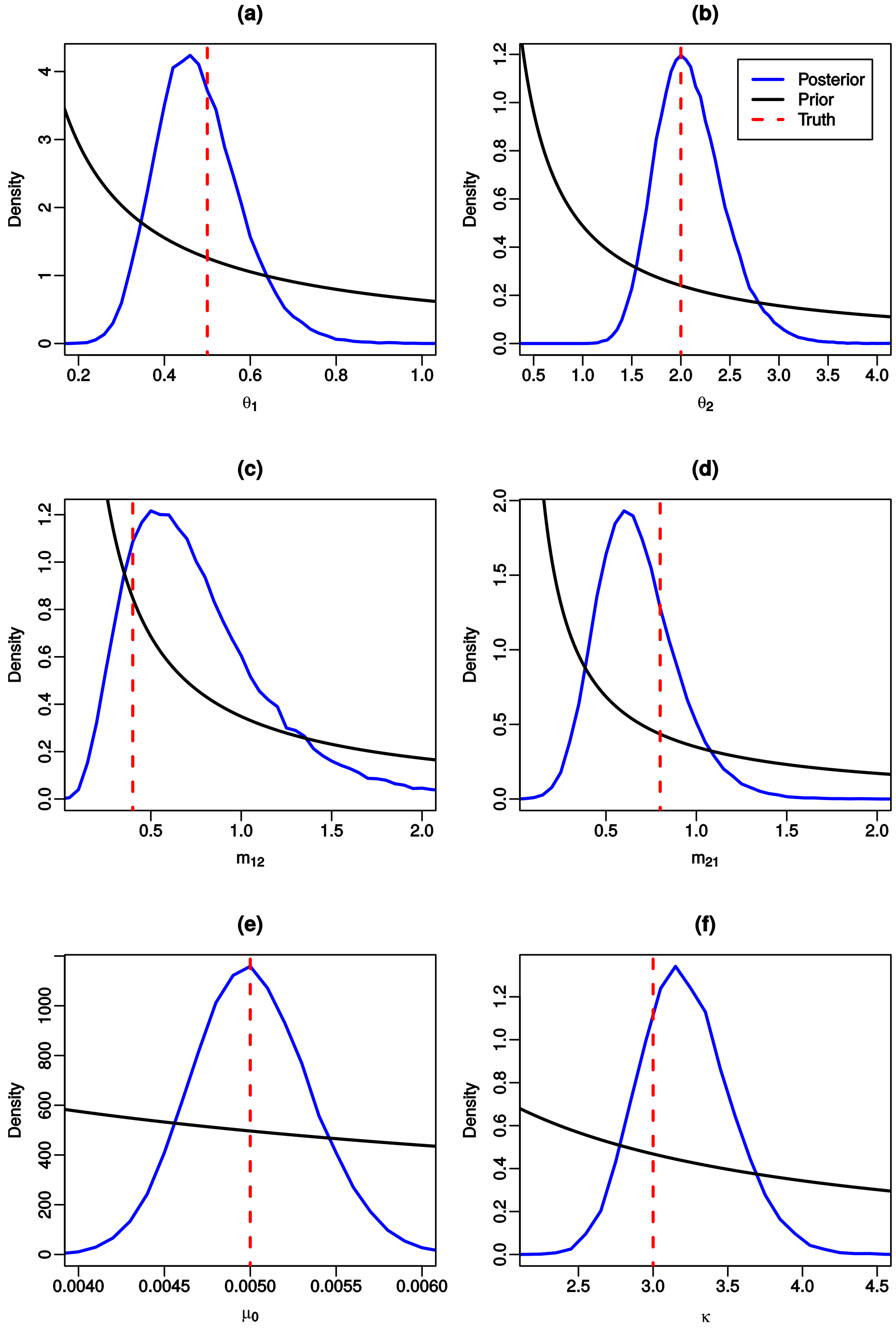

Figure 2: Sampled posterior densities obtained from a single simulated alignment generated under the 2 deme model, with (a) and (b) showing the population size estimates, (c) and (d) the immigration rate estimates and (e) and (f) illustrating the substitution model parameters. The dashed vertical bars mark the true value, while the black lines show the portion of the $\log \mathcal{N}(0, 4)$ prior used for each of the parameters (scaled vertically to improve clarity).

Model	Parameter	Truth	Median of posterior medians	95% HPD coverage
2 deme	θ_1	0.5	0.486	98%
	θ_2	2.0	2.02	93%
	m_{12}	0.8	0.841	95%
	m_{21}	0.4	0.379	97%
	μ_0	0.005	0.00497	97%
	κ	3	2.98	98%
3 deme	θ_1	0.5	0.484	94%
	θ_2	2.0	2.05	96%
	θ_3	1.0	1.05	87%
	m_{12}	0.4	0.410	95%
	m_{21}	0.2	0.161	92%
	m_{13}	0.4	0.360	93%
	m_{31}	0.2	0.169	94%
	m_{23}	0.2	0.207	96%
	m_{32}	0.4	0.312	92%
	μ_0	0.005	0.00493	92%
	κ	3	2.98	97%
4 deme	θ_1	0.5	0.504	97%
	θ_2	2.0	2.02	94%
	θ_3	1.0	1.01	90%
	θ_3	1.0	0.995	93%
	m_{12}	0.4	0.218	79%
	m_{21}	0.2	0.146	89%
	m_{23}	0.2	0.129	80%
	m_{32}	0.4	0.264	86%
	m_{34}	0.3	0.2144	91%
	m_{43}	0.3	0.240	88%
	m_{14}	0.4	0.316	90%
	m_{41}	0.2	0.096	95%
	m_{13}	0	0.0336	—
	m_{31}	0	0.0212	—
	m_{24}	0	0.0166	—
	m_{42}	0	0.0229	—
	μ_0	0.005	0.00498	96%
	κ	3	2.98	96%

Table 1: Summary of results obtained from the full 2, 3 and 4 deme analyses. Coverages shown in the Table 1 of the manuscript are averaged over population size vector and immigration rate matrix elements. Note that coverages are not calculated for the excluded transitions in the 3 deme model, as the log-normal prior explicitly excludes the truth here.

immigration rate matrix parameters.

The results of these analyses are given in Table 2, indicating that the sampler is certainly capable of performing parametric inference under models with larger numbers of demes, provided there is sufficient information present in the data.

6 Migrate-n posterior comparisons

In order to directly compare inference using the implementation of the new algorithm, we conduct an additional inference of the 2 and 3 deme simulated sequence data sets analyzed in section 4.2 of the manuscript. In this inference, however, we apply uniform priors to the elements of the immigration rate matrix m and the population size vector θ. Additionally, we refrain from estimating the clock rate μ_0 and the transition/transversion ratio κ and instead fix these to the known true values of 0.005 and 3, respectively.

Figure 3 compares the sampled posteriors for the demographic parameters obtained using Migrate-n 3.6.4 from a single 2 deme simulated sequence alignment with those obtained from the same alignment with the same parameter priors using the implementation of the algorithm described in the manuscript. (Both chains were run for 10^7 steps with a 10^6 step burn-in period.) This close agreement is strong evidence that we have implemented the structured coalescent model correctly, and that the operators described in our paper are not biasing the posterior. (The BEAST 2 and Migrate-n input files used to perform this comparison are provided in the supplementary file `MigrateComparisonScripts.zip`.)

Figures 4 and 5 compare the sample median values generated using each of the two methods (again with 10^7 steps) for all population size and migration parameters estimated from the 2 and 3 deme simulated sequence data-sets. While there is a degree of sampling noise, the agreement is extremely good.

We emphasize that while both Migrate-n and our package employ MCMC, these are two utterly independent implementations using algorithms that differ substantially in the details. Their agreement with one another is therefore extremely strong evidence that both methods have been implemented correctly.

Model	Parameter	Truth	Median of posterior medians	95% HPD coverage
5 deme	θ_1	0.5	0.454	95%
	θ_2	1	0.950	92%
	θ_3	1	0.929	92%
	θ_4	1	0.974	94%
	θ_5	2	2.03	95%
	$\bar{m}$	0.05	0.0487	87%
	μ_0	0.005	0.00493	96%
	κ	3	3.03	92%
10 deme	θ_1	0.5	0.473	94%
	θ_2	0.5	0.481	95%
	θ_3	0.5	0.493	93%
	θ_4	1	1.05	98%
	θ_5	1	1.03	99%
	θ_6	1	1.01	97%
	θ_7	1	0.986	95%
	θ_8	2	2.10	94%
	θ_9	2	2.00	94%
	θ_{10}	2	1.98	92%
	$\bar{m}$	0.01	0.0102	94%
	μ_0	0.005	0.00496	95%
	κ	3	3.03	96%

Table 2: Summary of results obtained from the constrained 5 and 10 deme analyses, where $\bar{m}$ represents the uniform migration rate between demes.

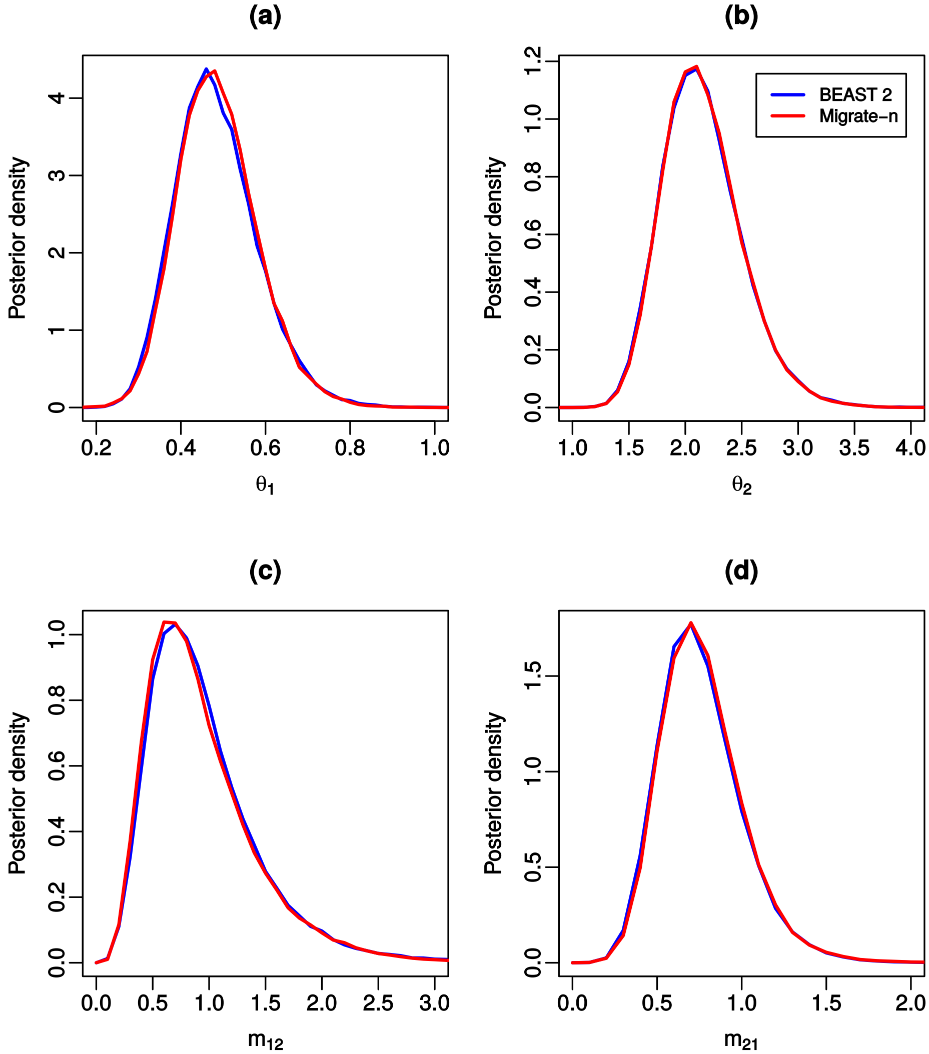

Figure 3: Comparison between sampled posteriors obtained by the new BEAST 2 structured coalescent sampler and those obtained using Migrate-n when applied to the a single alignment simulated under a 2 deme model. Sub-figures (a) and (b) show the scaled population size densities, while (c) and (d) display the immigration rate matrix elements.

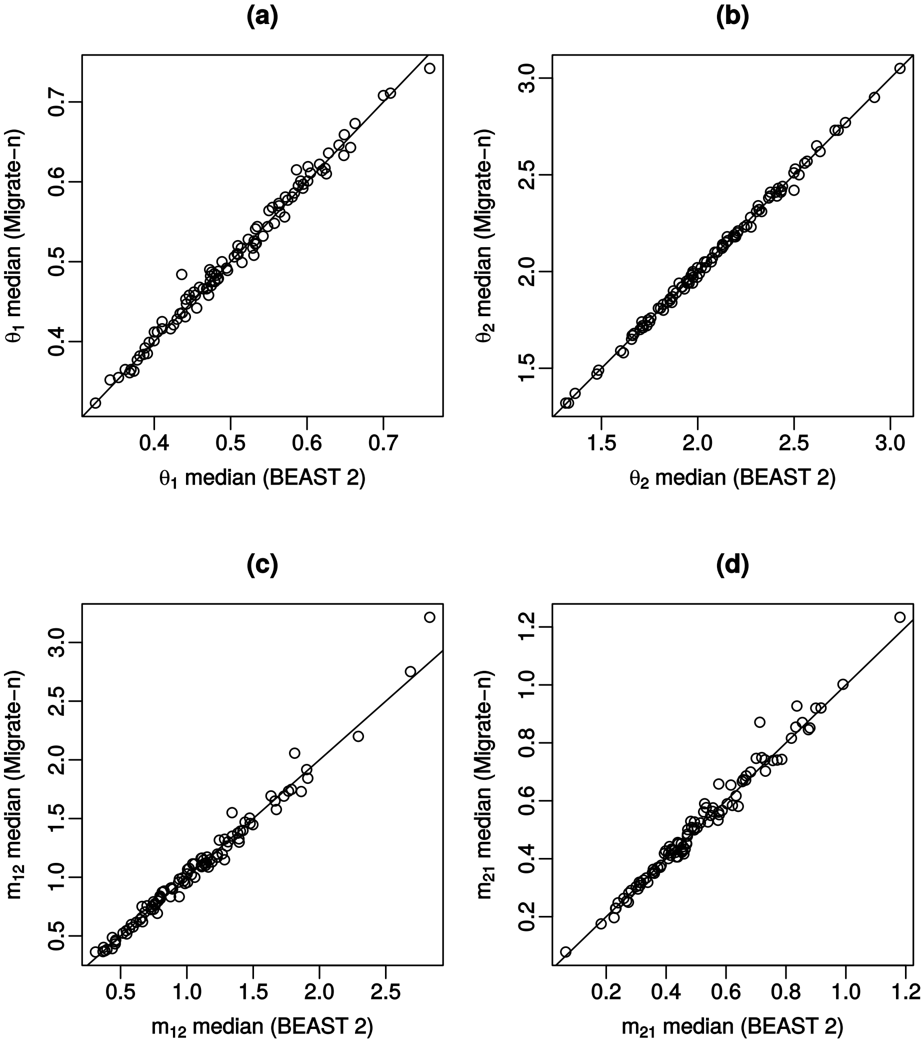

Figure 4: Comparisons between median posterior estimates of demographic model parameters from the 2 deme simulated sequence data set obtained using the BEAST 2 package developed here (horizontal axes) and Migrate-n (vertical axes). Sub-figures (a) and (b) compare the scaled population estimates while (c) and (d) compare the migration rate estimates. The diagonal lines represent perfect correspondence.

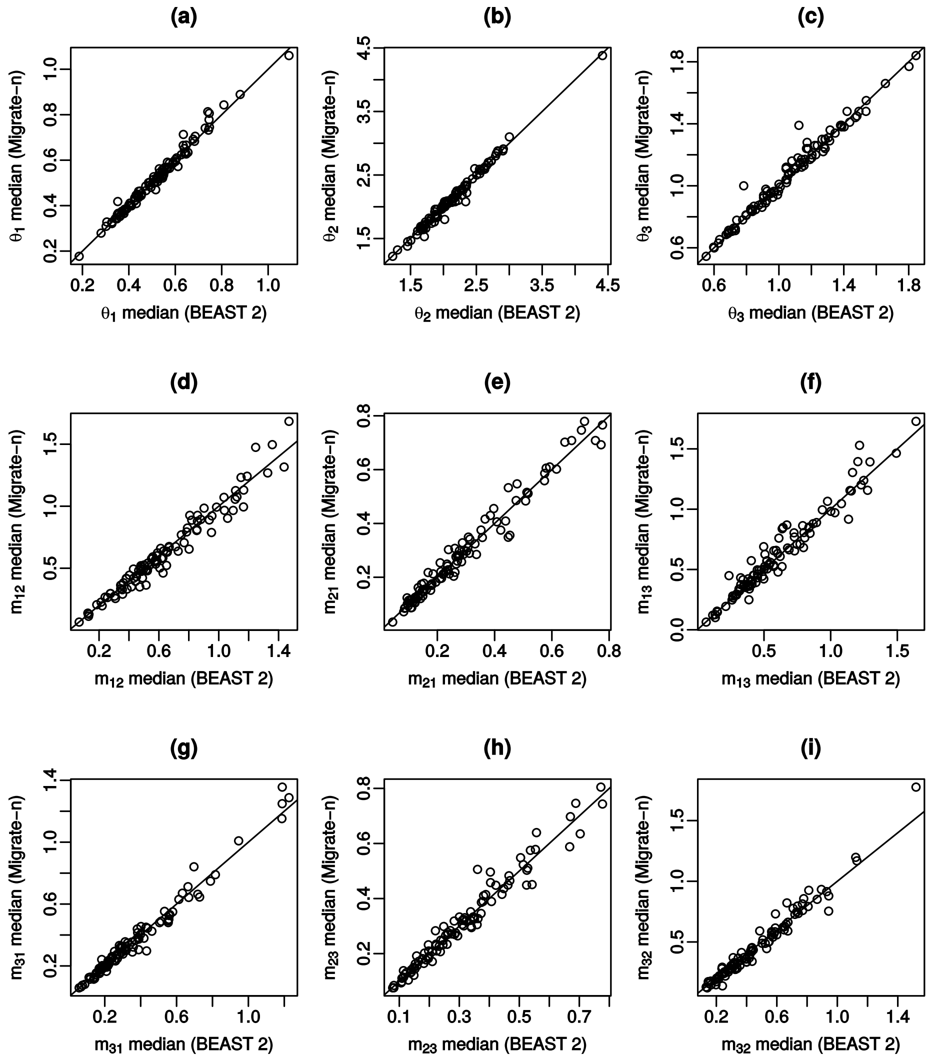

Figure 5: Comparisons between median posterior estimates of demographic model parameters from the 3 deme simulated sequence data set obtained using the BEAST 2 package developed here (horizontal axes) and Migrate-n (vertical axes). Sub-figures (a) through (c) compare the scaled population estimates while (d) through (i) compare the migration rate estimates. The diagonal lines represent perfect correspondence

7 H3N2 analysis

In this section we provide some details of the convergence testing and the full inference results for the H3N2 influenza analysis. Figure 6 illustrates the convergence of the distribution over the log posterior densities obtained from three independent MCMC chains, while Table 3 lists the parameter estimates (including 95% confidence intervals).

Both a CSV file containing the NCBI GenBank accession numbers for the sequences used and the BEAST 2 XML input file used to conduct this analysis are provided in the supplementary archive file `H3N2Analysis.zip`.

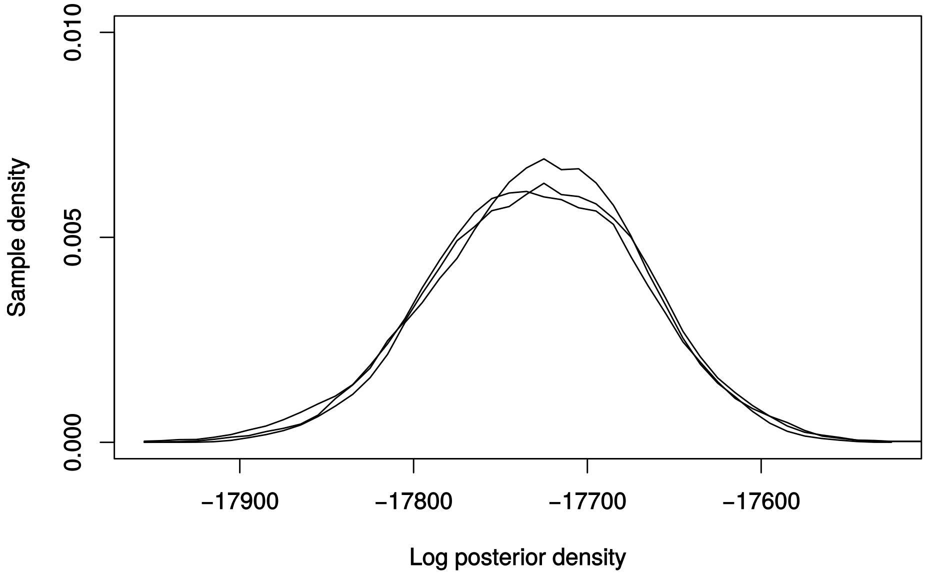

Figure 6: Sampled distributions of log posterior density for all model parameters (including structured tree) obtained from three independent MCMC chains for the H3N2 analysis. Close agreement indicates that burn-in size used (20% of the total chain length) was sufficient to attain convergence.

Parameter	Posterior median	95% HPD interval	ESS
θ_{NY}	1.67	$[1.33, 2.05]$	366
θ_{NZ}	0.864	$[0.687, 1.06]$	206
θ_{HK}	2.66	$[2.20, 3.15]$	376
$m_{\text{NY,NZ}}$	0.0614	$[0.000942, 0.192]$	483
$m_{\text{NZ,NY}}$	0.286	$[0.106, 0.516]$	586
$m_{\text{NY,HK}}$	0.270	$[0.115, 0.454]$	843
$m_{\text{HK,NY}}$	0.114	$[0.0187, 0.245]$	614
$m_{\text{NZ,HK}}$	0.592	$[0.242, 0.977]$	263
$m_{\text{HK,NZ}}$	0.133	$[0.00468, 0.368]$	178
Tree height	7.62 years	$[7.21, 8.11]$	724
Tree length	193 years	$[182, 205]$	144
Total migration count	85	[67, 106]	315
μ_0	5.03×10^{-3}	$[4.58 \times 10^{-3}, 5.51 \times 10^{-3}]$	353
$A \to C$ rate	0.210	[0.168,0.255]	27872
$G \to T$ rate	0.164	[0.126,0.205]	22339
$A \to T$ rate	0.0928	[0.0700,0.1175]	32291
$C \to G$ rate	0.0244	[0.0109,0.0409]	39570
$C \to T$ rate	1.21	[1.01,1.43]	16199
Gamma shape parameter	0.421	[0.364, 0.482]	63646

Table 3: Parameter estimates from H3N2 analysis, including . The "tree length", the sum of all branch lengths in the tree, mixes the most slowly. The parameters below the dividing line belong to the GTR+γ substitution model.

References

J. Felsenstein. Evolutionary trees from DNA sequences: a maximum likelihood approach. *J Mol Evol*, 17(6):368–376, 1981. URL http://www.ncbi.nlm.nih.gov/pubmed/7288891.

Joseph Felsenstein. *Inferring Phylogenies*. Sinauer Associates, Massachusetts, 2003.

Richard R. Hudson. Gene genealogies and the coalescent process. *Oxford Surveys in Evolutionary Biology*, 7:1, 1990.

M. Notohara. The coalescent and the genealogical process in geographically structured population. *J Math Biol*, 29(1):59–75, 1990. doi: 10.1007/BF00173909.

Philippe Lemey, Andrew Rambaut, Alexei J Drummond, and Marc A Suchard. Bayesian phylogeography finds its roots. *PLoS Comput Biol*, 5(9):e1000520, Sep 2009. doi: 10.1371/journal.pcbi.1000520. URL http://dx.doi.org/10.1371/journal.pcbi.1000520.

Wayne P. Maddison, Peter E. Midford, and Sarah P. Otto. Estimating a binary character's effect on speciation and extinction. *Syst Biol*, 56(5):701–710, Oct 2007. doi: 10.1080/10635150701607033. URL http://dx.doi.org/10.1080/10635150701607033.

Nicolas Rodrigue, Herv Philippe, and Nicolas Lartillot. Uniformization for sampling realizations of markov processes: applications to bayesian implementations of codon substitution models. *Bioinformatics*, 24(1):56–62, Jan 2008. doi: 10.1093/bioinformatics/btm532. URL http://dx.doi.org/10.1093/bioinformatics/btm532.

Alexei J. Drummond, Geoff K. Nicholls, Allen G. Rodrigo, and Wiremu Solomon. Estimating mutation parameters, population history and genealogy simultaneously from temporally spaced sequence data. *Genetics*, 161: 1307, 2002. URL http://www.genetics.org/content/161/3/1307.short.

Greg Ewing, Geoff Nicholls, and Allen Rodrigo. Using temporally spaced sequences to simultaneously estimate migration rates, mutation rate and population sizes in measurably evolving populations. *Genetics*, 168 (4):2407–2420, Dec 2004. doi: 10.1534/genetics.104.030411. URL http://dx.doi.org/10.1534/genetics.104.030411.

Timothy G Vaughan and Alexei J Drummond. A stochastic simulator of birth-death master equations with application to phylodynamics. *Mol Biol Evol*, 30(6):1480–1493, Jun 2013. doi: 10.1093/molbev/mst057. URL http://dx.doi.org/10.1093/molbev/mst057.

Jotun Hein, Mikkel H. Schierup, and Carsten Wiuf. *Gene Genealogies, Variation and Evolution: A Primer in Coalescent Theory.* Oxford University Press, USA, 2005.

Daniel T. Gillespie. A general method for numerically simulating the stochastic time evolution of coupled chemical reactions. *J. Comp. Phys.*, 22:403, 1976.
